# Influence of Vertical Soft Tissue Thickness and Keratinized Mucosa Width on Marginal Bone Loss Around Platform‐Matched and Platform‐Switched Implants: A Split‐Mouth Randomized Controlled Trial

**DOI:** 10.1155/ijod/2774110

**Published:** 2025-12-11

**Authors:** Le Trung Chanh, To Viet Thanh, Pham Nguyen Quan, Phan Dinh Nhat, Le Duc Lanh

**Affiliations:** ^1^ Department of High Technique, National Hospital of Odonto-Stomatology in Ho Chi Minh, Ho Chi Minh City, Vietnam; ^2^ Department of Prosthodontics, Faculty of Odonto-Stomatology, Hong Bang International University, Ho Chi Minh City, Vietnam; ^3^ Department of Implantology, Faculty of Odonto-Stomatology, Hong Bang International University, Ho Chi Minh City, Vietnam

**Keywords:** marginal bone loss, platform matching, platform switching, single implant, vertical soft tissue thickness

## Abstract

**Aims:**

To evaluate marginal bone loss (MBL) around implants restored with platform‐matched (PM) or platform‐switched (PS) abutments after loading and to examine the influence of vertical soft tissue thickness (STT) and keratinized mucosa width (KMW) on MBL.

**Materials and Methods:**

In a split‐mouth randomized controlled design of total 22 patients (44 implants), patients received one PM and one PS restoration in contralateral posterior mandibular sites. Standardized periapical radiographs were obtained immediately after restoration, at 3, 6, and 12 months to measure MBL. STT (thin, <2 mm vs thick, and ≥2 mm) and KMW (narrow, <2 mm vs wide, and ≥2 mm) were recorded. A linear multiple regression analysis was conducted with dependent variable (MBL) and independent variables (STT and KMW).

**Results:**

MBL did not differ significantly between PM and PS across follow‐up. In contrast, thin STT and narrow KMW were associated with greater bone loss over time. Multivariable analysis revealed that the soft tissue phenotype significantly influenced MBL, whereas the abutment design showed no significant effect (*β* = −0.43 for STT and *β* = −0.36 for KMW).

**Conclusion:**

Establishing a STT and KMW of ≥2 mm may be beneficial for bone preservation around implants, although further long‐term studies are needed for clinical practice.

## 1. Introduction

Maintaining bone stability and soft tissue health around implant is one of the prerequisites for long‐term success in implant treatment. The peri‐implant soft tissue phenotype is increasingly recognized as a key determinant of marginal bone stability [1, 2]. In clinical practice, two parameters are frequently discussed: vertical soft tissue thickness (STT) and keratinized mucosa width (KMW). Biologically, thicker tissues accommodate the peri‐implant biologic width more predictably, provide a better vascular supply, facilitate oral hygiene, and may dampen inflammatory challenge. Thin tissues, in contrast, are more prone to recession and early remodeling under function [1, 3, 4].

The literature, however, is not entirely consistent. Several clinical studies report greater peri‐implant inflammation and crestal remodeling when STT is thin or KMW is narrow, whereas others find little difference under excellent plaque control and carefully managed prosthetic contours. Research results have showed that marginal bone stability cannot be maintained if the peri‐implant soft tissue was thin, regardless of the type of implant‐abutment connection or the implant‐abutment‐to‐crest bone interface [2]. Recent studies have also pointed out the influence of STT and KMW on peri‐implant bone resorption [5–8]. Nevertheless, Canullo et al. [9] found that initial tissue thickness around the platform‐switched (PS) implant did not affect bone loss around the implant. Similar results from a controlled clinical trial of 30 patients conclude that there was no statistically significant difference of bone loss around implants in the thin tissue group versus thick tissue group [10]. This also concurs with studies of Longoni et al. [11] and Adibrad et al. [12]. The findings from a recent systematic review and meta‐analysis show that the impact of amount of KMW (either <2 mm or ≥ 2 mm) as a risk factor for developing peri‐implant disease remains low. Future control studies with proper sample size and longer follow‐up are needed to further validate current findings [13]. Much of this inconsistency likely reflects differences in how the tissues are measured (transmucosal probing, intra‐operative caliper, or ultrasound), the cutoffs used to define “thin” or “narrow” (commonly 2 mm), the follow‐up duration, and prosthetic variables such as emergence profile and abutment design [3, 12, 13].

Abutment platform configuration remains a controversy of bone preservation. Platform switching is intended to medialize the microgap and inflammatory cell infiltrate to protect the crest, yet clinical effects are often modest and may be modulated by soft tissue phenotype and prosthetic design. In some cohorts, PS appears beneficial; in others, its effect is small or difficult to detect once soft tissue and hygiene factors are optimized [14, 15].

These gaps justify a study design that limits between‐patient variability while observing implants during the phase most influenced by prosthetics and maintenance. A split‐mouth randomized controlled trial allows both abutment configurations to be tested within the same patient under comparable local and systemic conditions. Focusing on bone loss after restoration keeps attention on the clinical period when emergence profile, occlusion, hygiene access, and soft tissue phenotype are likely to matter most [2, 11, 14].

Therefore, for better control of confounding factors, the aim of this split‐mouth randomized controlled trial study is to evaluate the influence of vertical STT and keratinized mucosa height on MBL in two groups: platform‐matched (PM) versus PS abutments after 1 year follow‐up. The primary objective of this split‐mouth randomized controlled trial is to compare peri‐implant bone loss restored with PM and PS abutments over 12 months. The secondary objective is to assess the influence of STT (thin vs. thick) and KMW (narrow vs. wide) on MBL over time. The null hypothesis is that PS abutments would result in less bone loss than PM abutments. However, this advantage might be diminished in sites with thin STT or narrow KMW.

## 2. Materials and Methods

### 2.1. Participants and Sample Size

This split‐mouth randomized controlled trial enrolled 22 patients, each contributing two posterior mandibular implant sites (one per side), for a planned total of 44 implant sites. The study was conducted at the Department of High Technique treatment, National Hospital of Odonto‐Stomatology, Ho Chi Minh City, Vietnam, from December 2018 to April 2023. All patients voluntarily participated in the study. Selection and exclusion criteria followed the previous studies’ criteria that did not required bone graft in implant placement [1, 16]. The required sample size was estimated using the formula for comparing two related groups with repeated measures, with significance level (*α*) = 0.05, statistical power (1−*β*) = 0.80, correlation coefficient (*ρ*) between time points = 0.70, one pretreatment measurement and three posttreatment measurements. Based on the more conservative estimate from Lago et al. [17], the calculated effect size was 0.53, leading to a minimum of 18 participants per group. Allowing for a potential 10% dropout rate, the final sample size was adjusted to 20 participants per group.

### 2.2. Ethics and Registration

The study protocol was approved by the Ethics Council, University of Medicine and Pharmacy, Ho Chi Minh City, Vietnam (Number 443/DHYD‐HDDD). All participants provided written informed consent. This randomized controlled trial was conducted in accordance with the Consolidated Standards of Reporting Trials (CONSORT 2010) guidelines for reporting parallel and split‐mouth randomized trials [18].

#### 2.2.1. Selection Criteria

- Patients were over 18 years old with good health condition.
- Patients had missing lower molars symmetrically.
- Patients had stable intercuspation.
- Patients had sufficient bone for implant placement without the need of bone graft.
- Keratinized tissue covers the bone crest at least 5 mm.

#### 2.2.2. Exclusion Criteria

- Patients had general contraindications for oral surgery.
- Patients had systemic diseases that may affect osseointegration.
- Patients received radiotherapy in the head, face, and neck area within 12 months.
- Patients used drugs that affect bone metabolism.
- Patients had bruxism, temporomandibular joint disease, unstable psychology, and uncooperative.
- Patients who smoked more than 10 cigarettes/day.
- Patients were alcoholics, pregnant, or breastfeeding.
- Patients had progressive periodontitis in the maxillary remaining teeth or had an acute infection in the oral cavity.
- Patients will be excluded from the study if the torque was smaller than 35 Ncm during implant placement.

### 2.3. Research Methods

#### 2.3.1. Research Design

Double‐blinded, split‐mouth randomized clinical trial was conducted in this study.

### 2.4. Research Procedures

#### 2.4.1. Preoperative Assessment

**Figure 1:**
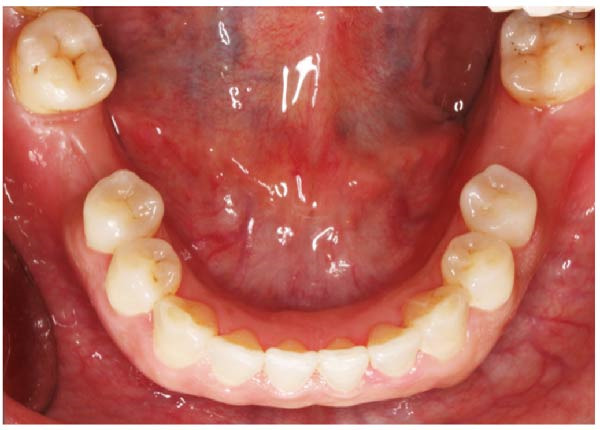
Initial status before implant treatment.

Primary impressions for making surgical guides, clinical assessment of the restoration space and soft tissue were conducted for each patient (Figure 1).

#### 2.4.2. Randomization and Allocation Concealment

**Figure 2:**
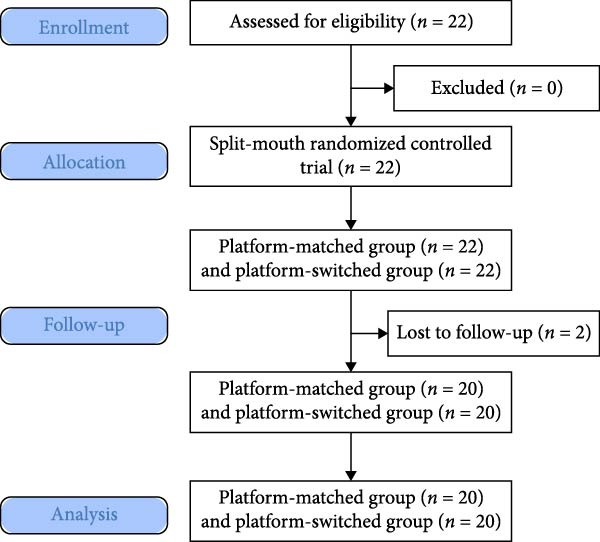
CONSORT 2010 flow diagram [18].

For each patient, the two implant sites (left/right) were allocated 1:1 to PM or PS abutment assignment using the website www.random.org. This process was prepared by an independent assistant who was not involved in treatment or outcome assessment. Allocation was concealed in sequentially numbered, opaque, sealed envelopes opened after osteotomy planning and site confirmation (Figure 2).

All patients underwent the same surgical procedure with one surgeon. The operating surgeon blinded to allocation until the site preparation was completed. Patients and radiographic outcome assessors were blinded to abutment assignment. Single crown was performed by a 5‐year experienced prosthodontist and clinical parameters were evaluated by a 5‐year periodontist.

#### 2.4.3. Implant surgery

**Figure 3:**
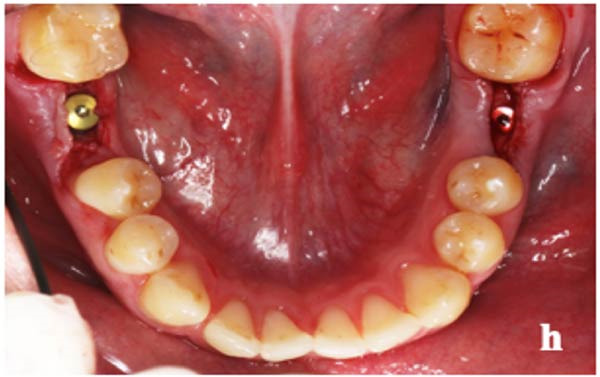
Placing healing abutment and suturing the elevated flaps.

Local anesthesia with 2% Lidocaine 1: 100,000 was performed at the surgical site. First, an incision was made on the ridge crest and full‐thickness flap was elevated to preserve maximum keratinized tissue on the buccal and lingual sides of the flap. The implant placement area was prepared with the surgical guide according to the manufacturer’s instructions, and the implant was placed in a one‐stage procedure. The bone drilling position was planned to ensure that the implant was placed in the center and the implant was placed under the bone crest. All sites received bone‐level titanium implants from Nobel Biocare, Switzerland, with Nobel Replace Tapered Groovy for PM group and Nobel Replace Platform Shift for PS group. Implant diameters were 3.5–4.3 mm and lengths 8–11.5 mm according to site anatomy. The implant was placed at bone level for PM implant group and 1 mm below bone level for PS implant group. The flap was closed with 5.0 silk suture (B. Braun Melsungen AG, Melsungen, Germany) (Figure 3). The patients were prescribed the following medicines: amoxicillin 500 mg; flagyl 250 mg, three times a day, one tablet each time, for 5 days; efferalgan codein, three times a day, one tablet each time, for 3 days. A 0.12% chlorhexidine solution Kin Gingival Complex (Laboratories KIN, Spain) was prescribed for mouth rinsing within 7 days. The suture was removed after 10 days after healing. Healing followed a two‐stage protocol with transmucosal healing abutments placed at second stage.

#### 2.4.4. Implant restoration

**Figure 4:**
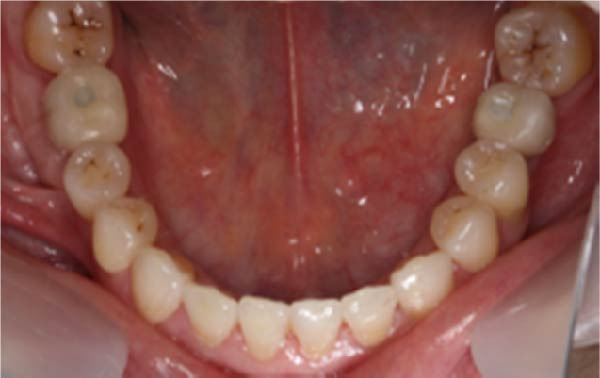
Final restoration.

**Figure 5:**
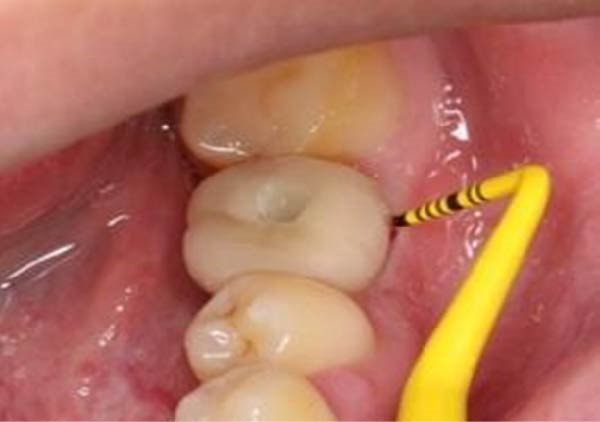
Measuring the depth of the gingival sulcus around the implant.

**Figure 6:**
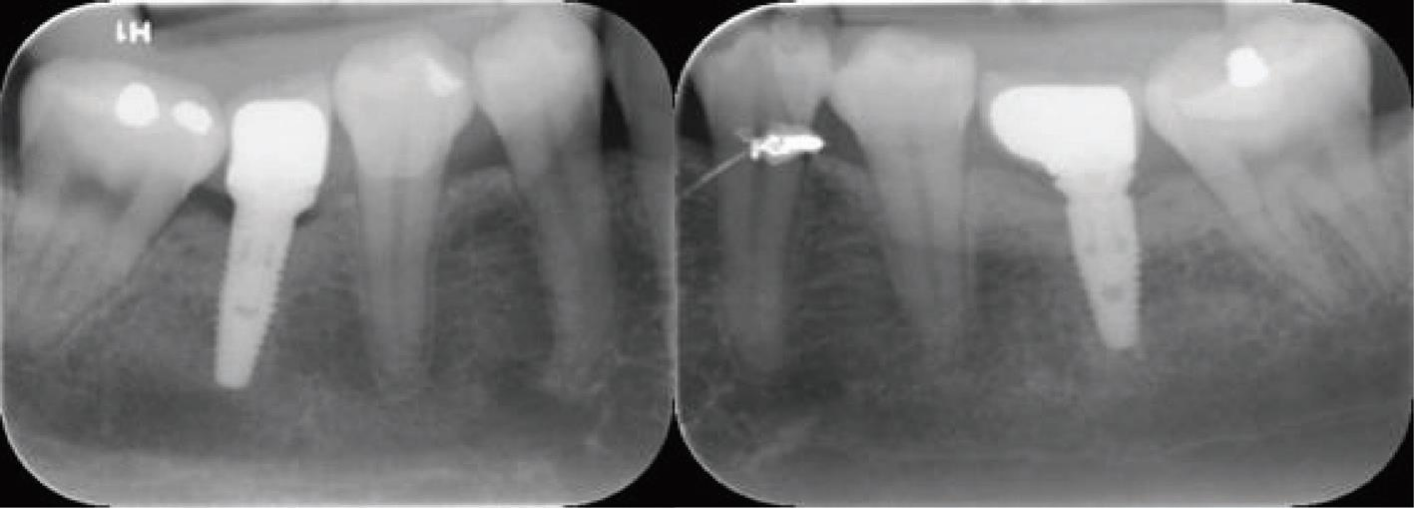
X‐rays at each point follow‐up time at 12‐month follow‐up.

After 3 months, metal–ceramic single crowns were delivered with either PM or PS abutments per randomization, with standardized emergence profile and occlusal scheme. Occlusal adjustments targeted light centric contacts and no excursive interferences. In this study, all patients had an opposing natural dentition to the implant. The definitive single‐crown restorations were fabricated by one prosthodontist (Figure 4, 5). X‐rays were taken immediately after the restoration was cemented and after 6 and 12 months (Figure 6, 7).

Figure 7Measurement of crestal bone change: PM group (a) and PS group (b).

**Figure:**
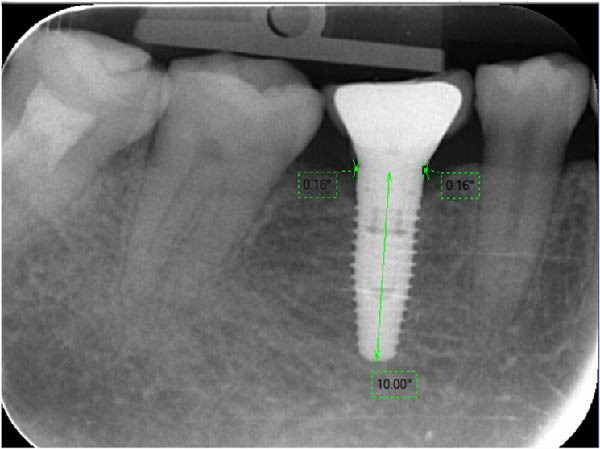
(a)

**Figure:**
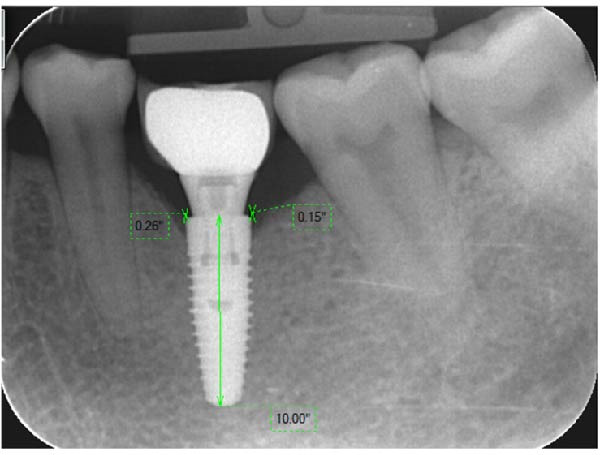
(b)

### 2.5. Evaluation of Implant and Prosthetic Success and Failure

Clinical and radiography outcomes were evaluated immediately after restoration (*T*_0_) and at 3 (*T*_3_), 6 (*T*_6_), and 12 months (*T*_12_).

#### 2.5.1. Evaluation of Vertical STT

**Figure 8:**
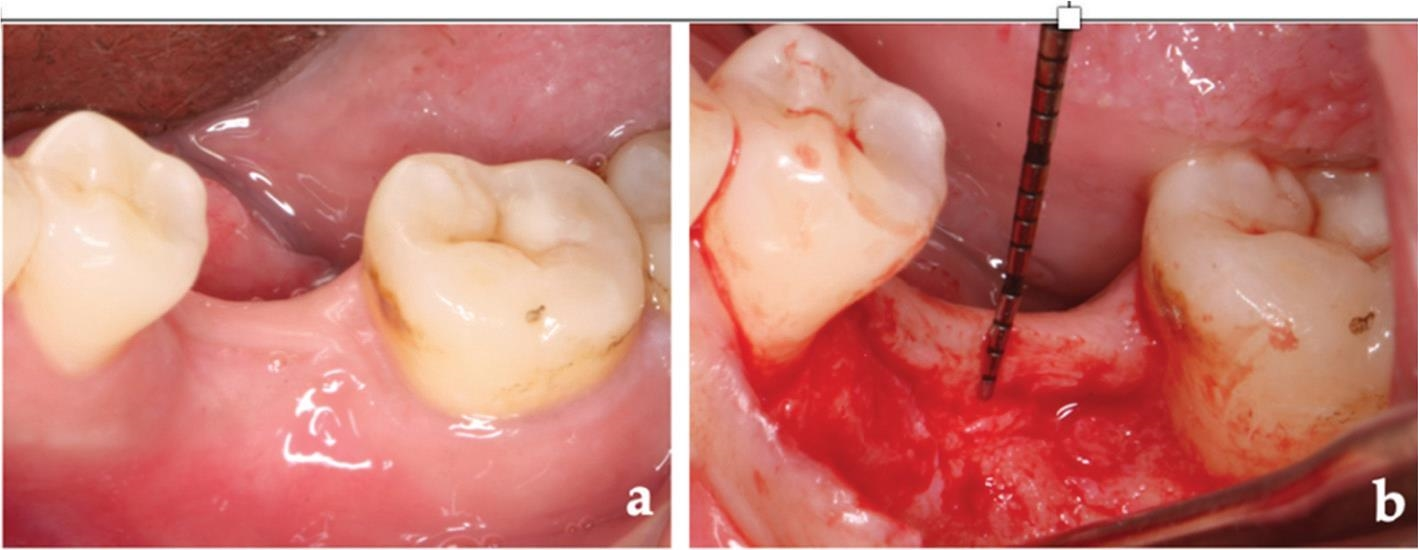
Measurement of soft tissue thickness in the mesio‐distal direction: (a) preoperative image; (b) measurement of thickness after lateral flap elevation.

To measure the STT at baseline, first, a full‐thickness flap was elevated on buccal side. STT was measured intraoperatively at the mid‐buccal aspect using a periodontal probe, rounded to 0.5 mm (Figure 8). KMW at baseline was measured from the mucogingival junction to the free mucosal margin at the buccal midline with the probe perpendicular to the mucosal surface (Figure 9).

Figure 9(a) Measurement of keratinized tissue height R36 (4 mm); (b) keratinized tissue height R46 (4 mm) on the same patient.

**Figure:**
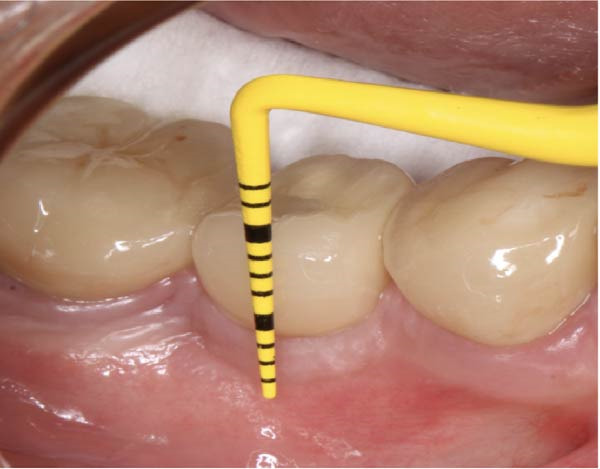
(a)

**Figure:**
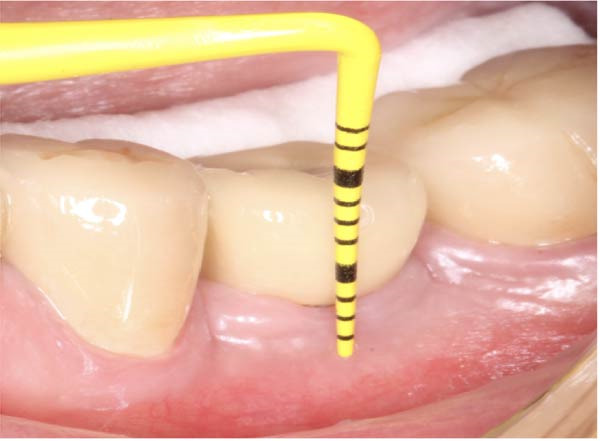
(b)

#### 2.5.2. Evaluation of the Marginal Bone Level on the Periapical Radiograph

**Figure 10:**
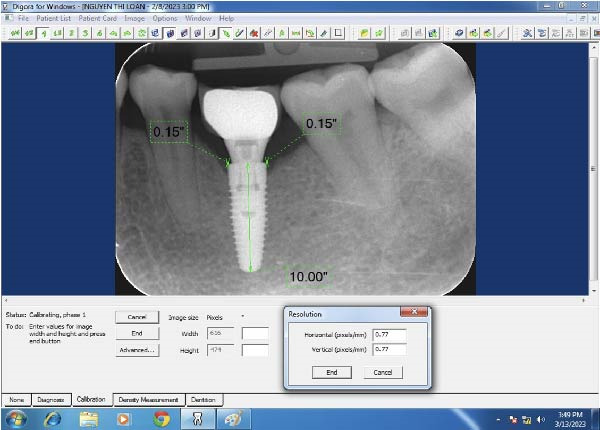
Digora software interface and resolution for size correction.

The marginal bone level was evaluated by digital intraoral radiographs using paralleling technique. The acceptable periapical radiograph was evaluated based on the clarity of the implant threads on the film. Each radiograph was calibrated based on the reference to the implant diameter (Figure 10). Digora software (Digora, France) was used for length measurement on the digital periapical radiograph based on the known implant. The measurement unit was in millimeters with decimal places to hundredths of a millimeter. The distance from the mesial and distal bone crest to the implant‐abutment interface horizontally (reference point) was presized on each film, the shortest thread pitch can be measured as 0.1 mm. The dimensions were measured mesial and distal to the implant axis and the average value was taken.

**Figure 11:**
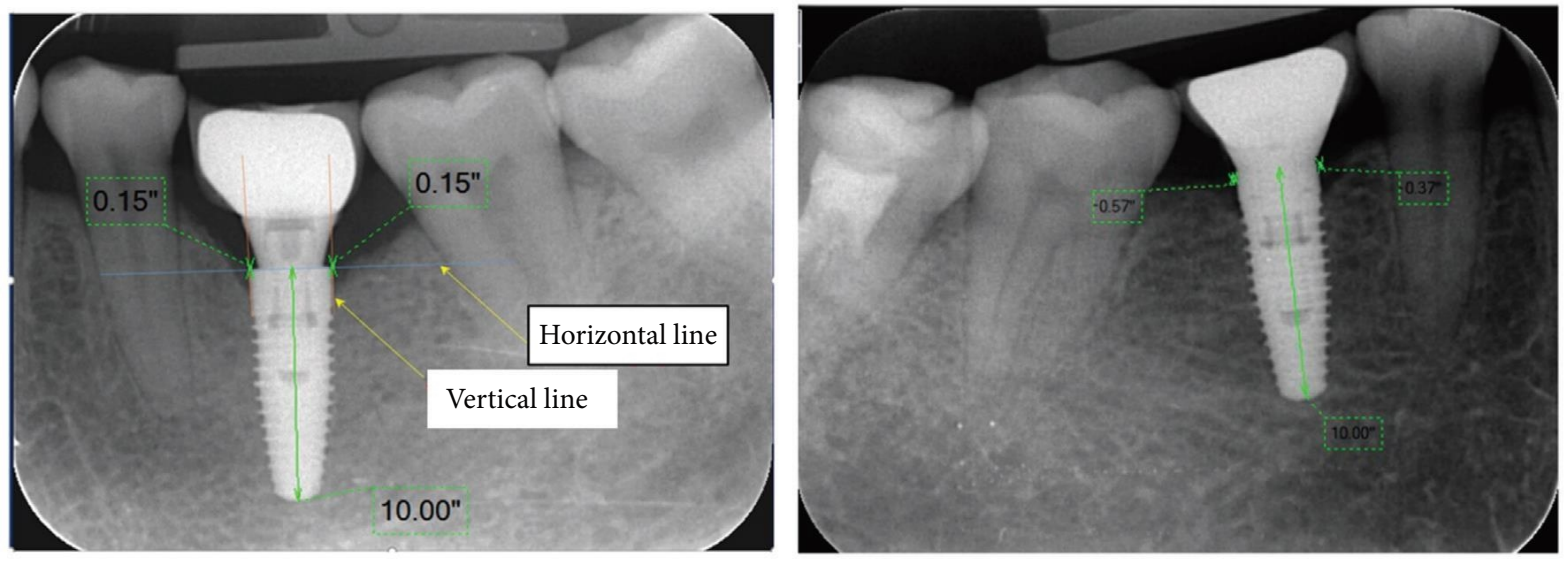
Measurement of the marginal bone loss.

The measurement method of marginal bone level was illustrated in Figure 11. First, a horizontal line was drawn at the shoulder of the implant and a vertical line at the center of the implant. The first bone contact with the implant was measured from the shoulder and both the proximal and distal sides of the implant were recorded [16]. The length on the periapical radiograph was measured from the center of the base to the tip of the implant. Peri‐implant bone loss was calculated by the following formula:

Peri‐implant bone loss = Marginal bone level (*T*_0_) − marginal bone level (*T*_3_/*T*_6_/*T*_12_).

All measurements were performed three times at each position by one calibrated examiner and the average value was used. To assess repeatability, 20% of images were remeasured ≥2 weeks later in random order, blinded to the first reads. The intraclass correlation coefficient (ICC) was 0.95, 95% CI (0.88–0.97) for mesial measurements and 0.91, 95% CI (0.78–0.96) for distal measurements.

### 2.6. Data Processing and Analysis

Implant and prosthesis success rates were compared between PM and PS groups. The average MBL (primary outcome), STT and KMW (secondary outcomes) were analyzed and compared between PM and PS groups using Mann–Whitney test. The mean difference with 95% confidence intervals were reported as effect size ± 95% CI for MBL at *T*_12_. Evaluation of the influence of relevant factors including implant length, bone density, mucosal thickness on MBL were analyzed using Spearman’s rank correlation. A linear multiple regression analysis was conducted with dependent variable (MBL) and independent variables (STT and KMW). Statistical tests were considered significant if the *p*‐value was less than 5%.

## 3. Results

### 3.1. Baseline Demographic and Clinical Characteristics

A total of 22 patients participated in the study; however, two patients did not return for examination as scheduled. The drop out ratio was 9.10%. The study was conducted on 20 patients with the average age of 45.21 ± 13.52 years old, ranged from 27 to 71 years old. Among that, nine patients were females (45%) and 11 were males (55%) (Table 1). No important harms or unintended effects were observed in either group during the trial or follow‐up.

**Table 1:** Baseline demographic and clinical characteristics.

Groups	PM group (*n* = 20)	PS group (*n* = 20)
Sex		
Female	11 (55)	
Male	9 (45%)	
Age (yrs old, mean ± SD)	45.21 ± 13.52	
Implant diameter		
3.5 mm	8	10
4.5 mm	12	10
Implant length		
8 mm	2	4
10 mm	6	8
11.5 mm	10	7
13 mm	2	1
Bone density (HU, mean± SD)	672.51 ± 252.91	663.11 ± 201.23
Vertical soft tissue thickness (mm, mean ± SD)	2.30 ± 0.64	2.57 ± 0.73
Keratinized mucosa width (mm, mean ± SD)	2.70 ± 1.10	2.80 ± 0.83

### 3.2. Implant and Prosthesis Outcomes

Over 12 months, there were no adverse events, implant failures, or prosthetic complications. All patients tolerated the procedures well, and the healing process was uneventful. The rate of vertical MBL around the implant was <1.5 mm, there was no periapical radiolucency, no pathological periodontal pockets, and all implants had good osseointegration. In addition, no prosthesis‐related complications occurred during follow‐up interval. The success rate of prosthesis was 100% in both PM and PS groups, with no between‐group difference (*p* = 0.09).

### 3.3. Bone and Soft Tissue Outcomes in PM and PS Groups

In PM group, STT, KMW, and MBL were 2.30 ± 0.64 mm, 2.70 ± 1.10 mm, and 0.34 ± 0.14 mm, respectively. In PS group, STT, KMW, and MBL were 2.57 ± 0.73 mm, 2.80 ± 0.83 mm, and 0.30 ± 0.26 mm, respectively (Table 2). There was no statistically significant difference in STT, KMW, and MBL between the two groups after 12‐month follow‐up (*p*  > 0.05).

**Table 2:** Average marginal bone loss, vertical soft tissue thickness and keratinized mucosa width of PM and PS groups after12‐month follow‐up.

Variables	Platform matching	Platform switching	*p*‐Value
Vertical soft tissue thickness (mm)	2.30 ± 0.64	2.57 ± 0.73	0.21
Keratinized mucosa width (mm)	2.70 ± 1.10	2.80 ± 0.83	0.72
Marginal bone loss (mm)	0.34 ± 0.14	0.30 ± 0.26	0.27

*p* < 0.05

### 3.4. Subgroup Analyses by Soft Tissue Phenotype

#### 3.4.1. Vertical STT

The average STT of thin group (<2 mm) and thick group (≥2 mm) were 1.89 ± 0.21 mm and 3.11 ± 0.40 mm, respectively. There was a statistically significant difference in two groups (*p* = 0.02). Table 3 showed that MBL did not differ between two groups at *T*_3_ and *T*_6_ (*p*  > 0.05), but at *T*_12_, the MBL was lower in the thick group (0.24 ± 0.15 mm) than the thin group (0.39 ± 0.23 mm, with *p* = 0.01). The effect size estimate was 0.15 mm (95% CI 0.03**-**0.27 mm), indicating greater MBL in the thin group.

**Table 3:** The difference of marginal bone loss between two groups of vertical soft tissue thickness.

Groups		Thin group (<2 mm) (*n* = 22)	Thick group (≥2 mm) (*n* = 18)	*p*‐Value
Average thickness (mm)		1.89 ± 0.21	3.11 ± 0.40	**0.02** ^∗^
3‐Month follow‐up (*T*_3_)	Mean ± SD	0.22 ± 0.12	0.13 ± 0.12	
	Median (Q1–Q3)	0.18 (0.13–0.30)	0.11 (0.03–0.23)	0.09
6‐Month follow‐up (*T*_6_)	Mean ± SD	0.29 ± 0.23	0.19 ± 0.15	
	Median (Q1–Q3)	0.28 (0.15–0.38)	0.18 (0.05–0.26)	0.09
12‐Month follow‐up (*T*_12_)	Mean ± SD	0.39 ± 0.23	0.24 ± 0.15	
	Median (Q1 – Q3)	0.40 (0.25–0.43)	0.23 (0.09–0.35)	**0.01** ^∗^

*Note:* Bold indicates a statistically significant result.

^∗^ *p* < 0.05

#### 3.4.2. KMW

The average KMW of narrow group (<2 mm) and wide group (≥2 mm) were 1.51 ± 0.01 mm and 3.22 ± 0.68 mm, respectively. There was a statistically significant difference in two groups (*p* = 0.01). Table 4 showed that showed that MBL did not differ between two groups at *T*_3_ (*p* = 0.09); but at *T*_6_ and *T*_12_, the MBL was lower in the wide group (both *p* = 0.02). The average MBL of narrow group was twice larger than that of wide group. The effect size estimate was 0.21 mm (95% CI 0.01–0.41 mm), indicating greater MBL in the narrow group.

**Table 4:** The difference of marginal bone loss between two groups of keratinized mucosa width.

Groups		Narrow group (<2 mm) (*n* = 11)	Wide group (≥2 mm) (*n* = 29)	*p*‐Value
Keratinized mucosa width (mm)		1.51 ± 0.01	3.22 ± 0.68	**0.01** ^∗^
3‐Month follow‐up *(T_3_)*	Mean ± SD	0.22 ± 0.15	0.15 ± 0.11	0.09
	Median (Q1–Q3)	0.23 (0.13–0.30)	0.13 (0.05–0.21)	
6‐Month follow‐up *(T_6_)*	Mean ± SD	0.37 ± 0.30	0.20 ± 0.13	**0.02** ^∗^
	Median (Q1–Q3)	0.33 (0.25–0.46)	0.21 (0.10–0.28)	
12‐Month follow‐up *(T_12_)*	Mean ± SD	0.47 ± 0.29	0.26 ± 0.14	**0.02** ^∗^
	Median (Q1–Q3)	0.39 (0.25–0.59)	0.28 (0.18–0.40)	

*Note:* Bold indicates a statistically significant result.

^∗^ *p* < 0.05

### 3.5. Factors Associated With MBL

Spearman correlations showed no significant association between MBL and implant length, implant diameter, or bone density (all *p*  > 0.05) (Table 5). In contrast, STT and KMW correlated negatively with MBL (*r* = −0.41 and *r* = −0.32, respectively; both *p*  < 0.05).

**Table 5:** Correlation of clinical factors and marginal bone loss.

Variables	Average marginal bone loss	
	*R* (Pearson correlation)	*p*‐Value
Implant length	0.03	0.44
Implant diameter	0.22	0.09
Bone density	−0.05	0.39
Vertical soft tissue thickness	−0.41	**0.01** ^∗^
Keratinized mucosa width	−0.32	**0.02** ^∗^

*Note:* Bold indicates a statistically significant result.

^∗^ *p* < 0.05

Table 6 shows the results of linear multiple regression analysis with dependent variable (MBL) and independent variables (STT and KMW). A statistically significant relationship between MBL and both STT and KMW were observed, with *F* (2, 37) = 7.4, *p* = 0.002, *R* ^2^ = 0.25. Both STT and KMW were independent negative predictors of MBL (β = −0.36 and β = −0.43, respectively; both *p*  < 0.05).

**Table 6:** A multiple linear regression analysis.

Variables	Multiple regression analysis results			
	*B*	*β*	95% CI	*p*‐Value
Vertical soft tissue thickness	−0.11	−0.36	−0.19–0.02	**0.01** ^∗^
Keratinized mucosa width	−0.09	−0.43	−0.15–0.03	**0.04** ^∗^

*Note:* Bold indicates a statistically significant result.

^∗^ *p* < 0.05.

## 4. Discussion

In this study, no between‐group difference in 12‐month MBL for PM vs PS implants was observed. This result is consistent with prior studies of Uraz et al. [ 19] and Attia et al. [20]. In 5–10 years, several meta‐analyses report smaller MBL with PS compared to PM (~0.3–0.6 mm) [21]. A modest sample and 12‐month follow‐up may be underpowered to detect a small PS benefit in our study. Moreover, abutment/restoration protocols (timing, connection torque, and transmucosal emergence) likely minimize microgap influences. Nevertheless, implants restored with PS and PM perform equally regarding clinical and radiographic outcomes [22].

The results showed that thinner vertical soft tissue was associated with greater MBL at 12 months. In fact, STT is considered to be a factor that directly affects peri‐implant bone resorption [4, 22]. Biologically, thicker peri‐implant mucosa may better accommodate the biologic width, provide richer vascularization, buffer inflammatory challenges, and facilitate plaque control. These mechanisms plausibly limit early remodeling. Contemporary reviews increasingly frame STT not only as an esthetic consideration but also as a protective factor for hard tissue stability, consistent with our finding [23]. Many previous studies have shown the important role of thick mucosal phenotype in preserving peri‐implant bone tissue [1–3, 24]. About the keratinized mucosal width, the results of this study showed that narrow KMW (<2 mm) presents higher MBL than wide KMW (≥2 mm) with an effect size of 0.21 mm (95% CI 0.01–0.41 mm). A wider keratinized band may improve patient hygiene access, dampen soft tissue micromotion under functional load, and sustain a more stable junctional epithelium, which supports marginal bone stability. This pattern coheres with clinical studies reporting greater MBL when KMW is <2 mm [7, 8, 14, 15].

Considering STT and KMW together helps clarify why some implants maintain marginal levels despite similar hardware and protocols. The results also suggested that both STT and KMW contribute additively to bone stability in posterior mandibular sites. These associations remain in correlation and multivariable models, where STT and KMW are independent negative predictors of MBL. A key aspect of the present study was the simultaneous evaluation of the influence of soft tissue characteristics on MBL. It was observed that greater MBL occurred in cases with thin vertical soft tissue and a narrow width of keratinized mucosa, regardless of whether PS or PM implants were used. These findings further support the role of peri‐implant soft tissue in influencing MBL [23]. From a tissue‐biology perspective, thin tissues may struggle to establish a stable biologic width without initial crestal remodeling, reduced vascularity and thinner connective tissue compartments can limit inflammatory control and repair, especially in posterior load‐bearing zones. Prior work indicates that thick phenotype provides more resilience against MBL than thin phenotype [9, 12, 23].

Nevertheless, the findings of this study should be interpreted with attention to safety and applicability. Across groups, no adverse events or prosthetic complications occur over 12 months. Thus, the benefit–harm balance between PM and PS is neutral within this time frame and setting. Clinically, the absolute phenotype effects are small but may be relevant in higher‐risk situations (thin tissues, narrow KMW, and high esthetic demands). The advantage of our study was the measurement method of vertical mucosa thickness. Tissue thickness was measured from the top of the alveolar ridge to analyze the thickness of the mucosa, epithelium and connective tissue. The results may be more accurate than studies using transmucosal probing [9]. Several alternatives could be used to measure tissue thickness were using CBCT or high frequency ultrasound with good reliability [25].

Several limitations of the study should be acknowledged. First, although the sample size was statistically adequate to detect differences in MBL, it remained relatively small. Second, the follow‐up period was limited to 12 months after restoration, which may not fully reflect long‐term bone remodeling processes. Third, while the split‐mouth design helped reduce interindividual variability, potential cross‐arch behavioral influences could not be completely eliminated. Finally, patient‐centered outcomes such as esthetic satisfaction and changes in peri‐implant mucosal color were not assessed, which may have provided a more comprehensive evaluation of treatment outcomes [26].

The findings of this study are applicable to partially edentulous adult patients requiring bilateral posterior mandibular implant placement. Clinical consideration should aim for thicker vertical mucosa and KMW ≥2 mm to limit early MBL, regardless of PM or PS selection. When phenotype is unfavorable, clinicians might prioritize soft tissue optimization alongside standard surgical–prosthetic protocols [27]. However, generalization should be approached with caution for patients presenting with systemic health conditions, inadequate oral hygiene, or compromised bone quality, as these factors were excluded from the study. Furthermore, outcomes may vary with the use of different implant systems or surgical protocols.

## 5. Conclusion

Within the limitations of this study, MBL was driven more by soft tissue phenotype than abutment platform at 12 months after restoration. Thick STT and wide KMW (≥2 mm) align with more favorable bone behavior, compare to thin STT and narrow KMW (<2 mm). These soft tissue features should be considered as treatment planning parameters to guide augmentation decisions, rather than relying on platform design.

## Conflicts of Interest

The authors declare no conflicts of interest.

## Funding

The authors received no specific funding for this work.

## Data Availability

The data that support the findings of this study are available upon request from the corresponding author. The data are not publicly available due to privacy or ethical restrictions.
